# Magnetic Hyperthermia for Cancer Treatment: Main Parameters Affecting the Outcome of In Vitro and In Vivo Studies

**DOI:** 10.3390/molecules25122874

**Published:** 2020-06-22

**Authors:** Vânia Vilas-Boas, Félix Carvalho, Begoña Espiña

**Affiliations:** 1 UCIBIO-REQUIMTE, Laboratory of Toxicology, Biological Sciences Department, Faculty of Pharmacy, University of Porto, Rua de Jorge Viterbo Ferreira, 228, 4050-313 Porto, Portugal; vfevilasboas@gmail.com (V.V.-B.); felixdc@ff.up.pt (F.C.); 2 International Iberian Nanotechnology Laboratory, Av. Mestre José Veiga, 4715-330 Braga, Portugal

**Keywords:** magnetic hyperthermia, cancer treatment, efficiency, parameters

## Abstract

Magnetic hyperthermia (MHT) is being investigated as a cancer treatment since the 1950s. Recent advancements in the field of nanotechnology have resulted in a notable increase in the number of MHT studies. Most of these studies explore MHT as a stand-alone treatment or as an adjuvant therapy in a preclinical context. However, despite all the scientific effort, only a minority of the MHT-devoted nanomaterials and approaches made it to clinical context. The outcome of an MHT experiment is largely influenced by a number of variables that should be considered when setting up new MHT studies. This review highlights and discusses the main parameters affecting the outcome of preclinical MHT, aiming to provide adequate assistance in the design of new, more efficient MHT studies.

## 1. Introduction to Magnetic Hyperthermia: Concepts and Terminology

The term “hyperthermia” refers to the local, regional, or generalized increase in body temperature, and it has a solid, long-lasting history in the annals of cancer management, either alone or in combination with other therapeutic approaches.

**Figure 1:**
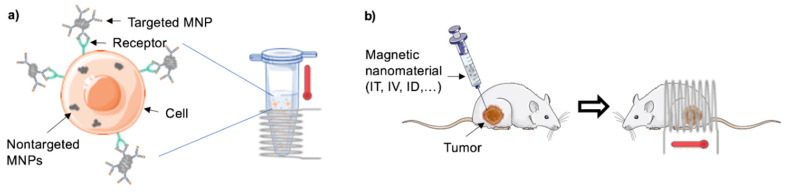
General schematic representation of a magnetic-hyperthermia study (**a**) in vitro and (**b**) in vivo. MNP—magnetic nanoparticle, administration routes: IT—intra-tumoral, IV—intravenous, ID—intradermal.

The use of magnetic implants as thermo-seeds to generate heat when exposed to an alternating magnetic field (AMF) has been proposed as cancer treatment since the 1950s [1]. The concept of “intracellular” magnetic hyperthermia (MHT) was later introduced by Gordon et al. by using dextran-coated magnetite submicron particles, which were internalized by cancer cells in vivo, to increase the temperature of tumors submitted to a strong AMF [2]. More recently, nanotechnology has been significantly contributing to the on-going scientific progress in the field of cancer research, not only regarding treatment but also its prevention and detection [3,4,5]. Innovative approaches have been proposed to treat cancer, among them magnetic nanoparticle (MNP)-induced MHT, which explores the heating ability of MNPs, under the influence of an AMF, to kill cancer cells (Figure 1). Some of the expected theoretical advantages of MHT are the possibility of treating a localized area, while keeping surrounding tissues safe, the ability to treat deep-seated tumors that would otherwise be untreatable and the possibility of exploring combinatorial schemes with other therapeutic regimes for increased efficiency [6].

Regardless of the clinical interest and the large availability of scientific literature on the topic, MHT for clinical treatment is hardly a reality and the number of preclinical studies progressing to clinical trials is minimal. Establishing comparisons between MHT studies is a challenge due to the widely different conditions used by different authors, which, in some cases, is aggravated by the lack of crucial information concerning a certain aspect of the procedure. In this review, the main parameters influencing the outcome of preclinical MHT studies will be addressed and discussed with the purpose of providing a source of helpful information for planning forthcoming MHT studies.

## 2. Main Parameters Influencing the Outcome of a Preclinical Magnetic Hyperthermia Study

The approval of nanomaterials for cancer clinical trials requires initial testing of their anti-cancer potential in vitro and in vivo in order to have an initial impression on the efficacy, tolerability, and toxicity of the MNPs and the selected AMF conditions. In general terms, an MHT experiment comprises three distinct layers: a biological component (cellular and/or animal models), a nanomaterial component (most commonly, MNPs), and an AMF component (considering the MHT equipment and the derived AMF specifications). A fourth level concerns the evaluation of the treatment outcome.

Some parameters will crucially impact the observed results, justifying the observed colossal differences in the outcome of MHT studies. Among those parameters, we highlight the cells to be tested, the MNPs’ characteristics (namely, the coating employed, the size, and initial concentration), the targeting system (if applicable), the selected animal model (if applicable), the AMF parameters, and the temperature that was reached. The evaluation of the MHT effect, i.e., which kind of test to perform, and the most appropriate time to implement it, also influence the reported outcomes. Table A1 and Table A2 compile examples of in vitro MHT studies, using non-targeted and targeted nano-formulations, respectively, while Table A3 concerns in vivo studies. The most relevant parameters that may have contributed to the observed outcome are summarized and will be further examined and discussed in this review.

### 2.1. The Biological Component—Cells, Cell Lines, and Animal Models

#### 2.1.1. Relevance and Thermal Susceptibility

Since MHT is often purposed for cancer treatment, it is important to test its tolerability and its efficiency to kill cancer cells that are representative of a tumor, both in vitro and in vivo. These are usually human cell lines that need to be tumorigenic to generate a tumor in an animal model. For some types of cancer, e.g., for glioblastoma, there are a number of different human cell lines that fill in this pre-requisite. However, the results of MHT studies often lead to distinct outcomes. In fact, different cells or cell lines may have diverse susceptibilities to heat, i.e., thermotolerance [7], which means that similar temperature profiles may lead to varying outcomes depending on the tested cells [8]. One major contributing factor for such differences may be the divergent induction of heat shock protein (HSP) synthesis among cell lines [9]. Some authors are actively trying to overcome this issue by blocking HSP70 to enhance MHT efficiency [10].

#### 2.1.2. Cell Number, Configuration, and Tumor Size

Since cancer cells tend to grow fast, the initial number of cells used for the experiments will have an impact on the observed results. An increased number of cells is able to uptake a larger total amount of magnetic material and, consequently, generate more heat when subjected to AMF. This means that using different number of cells might result in a different concentration of magnetic materials with all the associated consequences. This further applies to an in vivo scenario, as the number of tumorigenic cells inoculated in the animal model will result in variable tumor sizes.

The configuration in which the cells are exposed to MNPs and subjected to MHT may also result in different outcomes. In fact, under similar thermal loading, adherent cells seem to be more susceptible to MHT than suspended cells [11]. While in a cell suspension, the heating effects may be mediated by heating the surrounding medium, the real-time follow-up of the treatment on adherent cells revealed the delayed-onset of cell death occurring on a cell-by-cell basis, which, therefore, supported an intracellular type of MHT.

Other conformations of cells have been used for MHT studies like cell pellets [12] and cell clusters [13]. These cell clusters helped understand the influence of the tumor size on the efficiency of MHT, concluding that a minimum tumor volume of 1 mm^3^ is required for cytotoxic hyperthermia [13]. This result suggests the inability of MHT to treat microscopic tumors or metastasis.

**Figure 2:**
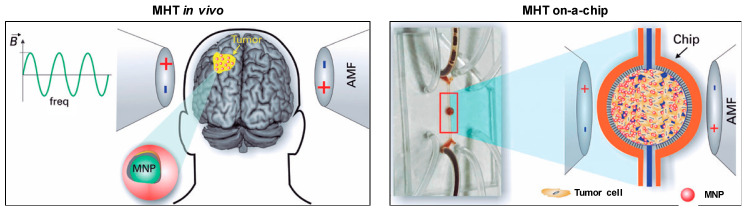
Schematic comparison between magnetic hyperthermia (MHT) in vivo and MHT on a tumor on-a-chip. In vivo, magnetic nanoparticles (MNPs) injected into the tumor are heated up by an alternating magnetic field (AMF). On the tumor-on-a-chip model, MNPs are injected using microfluidics into the central compartment where a 3D tumor structure was formed. The whole chip is then submitted to the AMF. Adapted with permission from Reference [15]. Copyright 2019 Instituto Israelita de Ensino e Pesquisa Albert Einstein.

In an attempt to better emulate the complex 3D structure of a tumor in vitro, Stocke and co-workers developed a co-cultured spheroid system containing triple negative breast cancer cells, endothelial cells, and embryonic fibroblasts from murine origin [14], which recreated a secondary lung tumor of a metastatic breast malignancy. After 10 days in culture (600 µm in diameter), these 3D structures were exposed to a low or a high concentration of inhalable MNPs and to AMF for 1 h, which resulted in a dose-dependent increase in cell death. The reached temperature was not reported and the contribution of heat for the observed outcome was not excluded, but the authors suggest it to derive from mechanical deterioration. Very recently, Mamica et al. proposed a new in vitro model consisting of a tumor-on-a-chip to study the effects of MHT on treating glioblastoma (Figure 2) [15]. The tumor was mimicked by using rat glioblastoma cells in 3D configuration in a device integrating microfluidics that guarantees the delivery of nutrients to the tumor. Future developments would include the emulation of the blood vessels in this model.

Ethical constraints impose a limit on the bearable tumor weight/volume for the experimental animal, but most of the in vivo MHT studies report initial tumor volumes ranging from 50 mm^3^ [16] to 1.4 cm^3^ [17] depending on the experimental animal model (Table A3).

#### 2.1.3. Animal Models for In Vivo Studies

The animal models mostly used for MHT studies are rodents, in particular, mice (Table A3). Yet, the efficiency of MHT has also been tested in bigger animals, such as rabbits [17], which can accommodate larger tumors. In many cases, the tumors are generated from xenographs of human cell lines, which is a procedure that requires immunocompromised animals. However, mouse cells can also generate the tumors, and, in this situation, immunocompetent mice may be used to gain insights into the role of anti-tumor immunity in the MHT treatment [18,19]. In fact, there is evidence of intense immuno-stimulation, in particular CD8^+^ T-cells recruitment and activation, following mild MHT treatment, which may contribute to increased efficiency of the treatment [19].

#### 2.1.4. Parallel Tests in Normal Cells

There are indications of the existence of distinct thermal susceptibility between normal and cancer cells [20,21]. However, this is only true for a certain range of temperatures and it does not exclude the occurrence of side effects mediated by nanomaterials (NMs). Therefore, it is important to test the proposed in vitro MHT strategies in normal cells to understand whether secondary effects should be expected when transitioning to in vivo studies. Only a minority of the studies mentioned in this review actually tested this (see Table A1 and Table A2). Fibroblasts, either from mouse origin [22,23,24,25] or from human [26,27,28] origin, are commonly used for this end. For targeted MHT studies, it may be more useful to use a cell line with a lower or even negative expression of the selected target, as this allows to simultaneously prove the selectivity of the treatment [21,25]. This type of study is particularly relevant when the selected target is also widely expressed in normal cells. As an example of the relevance of taking this type of study, Liao et al. functionalized MNPs with D-Galactosamine (D-Gal) to specifically target liver cancer cells, which results in very low levels of cell viability [29]. Even though the selected target, asialoglycoprotein receptor, is known to be widely expressed in normal liver cells as well [30], the authors did not reproduce the test in normal cells and, therefore, the occurrence of cytotoxicity in normal cells cannot be ruled out.

### 2.2. The Nanomaterial Component

#### 2.2.1. Size, Coating, and Chemical Composition

The size, size distribution, and shape of a particular NM have a clear impact on its magnetic properties [31]. These parameters should ideally be optimized [32] so as to exhibit the highest heating power under a selected AMF frequency [33,34,35], while displaying minimal toxicity if not subjected to an external magnetic field. This optimization also minimizes the need to revise extrinsic parameters, such as the NM concentration or the AMF power.

For MNPs, there is a critical size below which a superparamagnetic behavior is observed. Under an external magnetic field, the atomic magnetic moments of superparamagnetic nanoparticles align along the field direction, which achieves a high magnetic susceptibility [36]. Unlike other types of magnetic materials, once the magnetic field is removed, superparamagnetic nanoparticles behave like a non-magnetic material exhibiting no magnetic memory (i.e., no remanence), which yields stable and very useful colloidal dispersions for biomedical applications [37]. Additionally, the MNPs’ size also has clinical and in vivo significance. In fact, MNPs sized above 200 nm are rapidly taken up by the reticulo-endothelial system (RES) and accumulate in the liver and spleen while MNPs sized below 6 nm are filtered by the kidney [38]. Furthermore, there are natural differences between the neovasculature of tumors (defective and leaky) and that from normal tissues with the openings of normal vessels being generally less than 10 nm [39]. These differences pose practical implications since MNPs sized above 10 nm can extravasate and accumulate in the tumor, but not in the normal tissues. Additionally, the slow venous return and lower lymphatic clearance, which are characteristic of tumors, favor MNPs’ retention at the tumor site [40]. This extensive leakage and low clearance characteristic of many solid tumors are known as the enhanced permeability and retention (EPR) effect [39]. This seems, however, to fail in the clinical context [41]. At a cellular level, the upper size limit consensually considered for nanoparticle uptake through endocytosis is 500 nm [38,42]. As already mentioned, nanoparticles sized above 200 nm are generally taken up by specialized cells of the RES through phagocytosis, which is a specific type of endocytosis [43].

Very distinct types of magnetic materials have been used for MHT purposes, including metal nanoparticles (e.g., Fe, Co, and Ni), metal alloy nanoparticles (e.g., FeCo, FePt, CoPt, and FePd), metal oxide nanoparticles (e.g., Fe_3_O_4_, Fe_2_O_3_, and MnO), ferrite nanoparticles (e.g., MnFe_2_O_4_, NiFe_2_O_4_, and ZnFe_2_O_4_), metal-doped iron oxide nanoparticles (e.g., Mg, Mn, and Zn doped iron oxide), and core-shell magnetic nanoparticles (e.g., Fe@Fe_3_O_4_, Co@Co_2_P, and CoFe_2_O_4_@MnFe_2_O_4_) [44]. Since the magnetic properties of the NMs depend on their size, shape, composition, and structure, these characteristics need to be crucially controlled during NMs synthesis [32]. As an example, magnesium-doped maghemite superparamagnetic nanoparticles with 100× higher heating power (see Section 2.3.1) than the commercial Resovist formulation allowed the induction of complete necrosis of glioblastoma cells by applying a low AMF (*Hf* product = 1.22 × 10^9^ A m^−1^ s^−1^) [45]. However, using the same cellular model but applying a more extreme AMF (*Hf* product = 12.3 × 10^9^ A m^−1^ s^−1^), the use of lipid-based magnetic nano-vectors resulted in lower MHT efficiency (~50% apoptotic cells 72 h after 4 cycles of MHT) [46]. Still, these nano-vectors are rather versatile tools that can be loaded with chemotherapeutic agents to improve the treatment outcome.

The Curie temperature (Tc) is an intrinsic characteristic of the magnetic NM that depends on the NM’s composition. It is described as the temperature above which the materials become paramagnetic, i.e., the magnetism is lost and the heating stops [47]. This self-regulation of the system’s temperature by manipulation of the NM’s Tc has been suggested to maintain the temperature of the system within the hyperthermal range [48] in order to decrease the risk of overheating and consequent damage of neighboring (normal) tissues.

**Figure 3:**
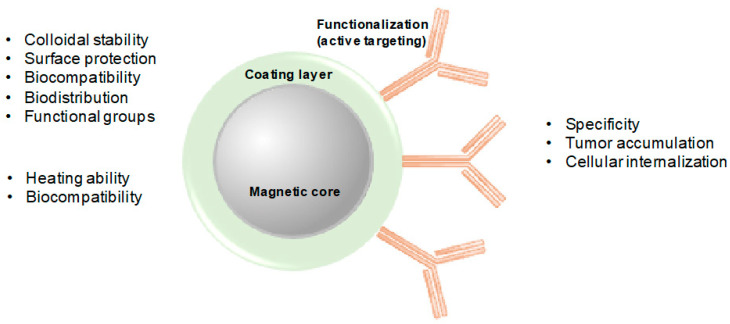
Simplified representation of a magnetic nanoparticle and its layers. Some of the factors affected by each magnetic nanoparticle (MNP) layer in in vitro and in vivo context are highlighted in the respective color text box.

The coating is a major contributor for NMs’ stability with significant repercussion on their magnetic properties as well as internalization ability and biocompatibility [49], which allows the application of NMs for nanomedicine purposes in general [31]. The coating materials protect the MNPs from oxidation, humidity, and acidity, and create a hydrophilic environment that prevents agglomeration, while allowing for further functionalization (Figure 3). Additionally, coating can act as a biocompatible shield for the MNPs, which may prevent their opsonization by the RES, consequently, increasing their blood circulation time [50]. Polymers, such as poly(ethyleneglycol) (PEG), poly(lactic-co-glycolic acid) (PLGA), alginate, dextran, and chitosan, are examples of commonly used polymeric stabilizers. Organic non-polymeric stabilizers can also be used for this end, namely oleic acid, stearic acid, and citric acid [51]. More details on the properties of NMs for MHT can be found in recent reviews [31,34,44].

#### 2.2.2. Targeting

The overexpression of some surface-receptors in cancer cells compared to normal cells allows for actively targeting cancer cells with MNPs functionalized with a targeting molecule such as an antibody, a peptide sequence, or a ligand protein [52]. This is in contrast with the passive targeting achieved with the EPR effect. The active targeting may result in efficient internalization of targeted MNPs by receptor-mediated endocytosis [53], and it has been described as one of the main factors affecting the binding of MNPs to cells in vitro [54]. The protein adsorption layer (protein corona), formed by the proteins in the medium where nanoparticles interact with cells (e.g., blood proteins in vivo, serum proteins in vitro), is known to contribute to lower adhesion, and, consequently, result in lower uptake. Yet, if a targeting moiety is present on the nanoparticles, the adhesion of the nanoparticles to the cells will fully depend on the target recognition [55]. In this sense, the preferential accumulation (and potential internalization) of targeted MNPs in cancer cells provides a controlled strategy to kill cancer cells with minimal predicted effects in normal cells. Even though recent studies support the superiority of targeted versus non-targeted MHT, both in vitro and in vivo [56,57], others defend that the amount of MNPs delivered by active targeting is insufficient to generate adequate heating at the tumor site [51]. Improvements to targeted MHT efficiency can be achieved by combining it with other therapies [26,57,58,59,60]. The authors preferred to explore MHT in a mono-therapeutic context while combining targeted with non-targeted MNPs for increased efficiency [21,61]. By adjusting the amount of non-targeted nanoparticles as a magnetic boost for the targeted nanoparticles, it is possible to push up the temperature to the level necessary to induce consistent cancer cell death rates. This strategy was highly effective in vitro, without the need to resort to highly cytotoxic, non-selective, chemotherapeutic agents.

Examples of regularly targeted molecules are the folate receptor and the human epidermal growth factor receptor 2 (HER2) receptor. While the folate receptor was found to be overexpressed in a broad variety of cancers and minimally expressed in normal tissues [62], the HER2 receptor is specifically expressed in some aggressive types of breast cancer [63].

#### 2.2.3. Concentration, Time of Incubation with the Cells, and Nanomaterial Excess Removal

The amount of NM and the time that it is incubated with cells prior to the AMF application usually positively correlate with the uptake of magnetic material, often expressed as pg Fe/cell, which, in turn, contributes to the reached temperature and consequent toxic effects. Comparatively to normal cells, cancer cells seem to display an improved ability to uptake NMs [21,64,65,66,67]. Even though this behavior cannot be generalized to other cell types rather than the studied ones, it appears as a natural mechanism favoring NMs’ accumulation in cancer cells.

It seems hard to establish the Fe loading necessary to achieve efficient MHT, but some studies tried to address this issue. A cancer cell suspension containing a concentration of 2–4 g Fe/L has been reported to generate temperatures between 40 and 45 °C, which causes a 50% drop in cell viability, while 5 g Fe/L resulted in 65 °C and total cell death [68]. However, using high concentration of NM for the MHT tests increases the chances of observing secondary effects, particularly in the in vivo context. A study has suggested that a minimum of 250,000 cells, each loaded with 200 pg of Fe, is required to reach 41.3 °C [13]. Considering the final volume of 0.5 mL, this represents a concentration of 0.1 g Fe/L, which is far below the ones from the previous study. In line with this result, the authors’ work on in vitro MHT using a combination of targeted and non-targeted MNPs reported temperatures of 43 to 47 °C and cell viability levels below 20% for cancer cells with a total iron load of 0.31 [61] and 0.65 g Fe/L [21]. When normal cells were treated under similar conditions, a total Fe loading of 0.2 g Fe/L was achieved and the temperature was kept around 41 °C with minimal impact on cell viability [21]. The single cell iron loading for those studies may be found in Table A2. Others have reported elevated MHT efficiency using very low concentration of the luteinizing hormone—releasing hormone (LHRH)-targeted MNPs [69]. The cells containing ca. 15 pg Fe/cell were heated up to 44 °C, which resulted in 5% cell viability. This is a low Fe content when compared with the study by Liao et al. that reported similar viability results but with ca. 365 pg Fe/cell [29]. This discrepancy may result from distinct thermal susceptibility of the cell lines or the different number of cells used in the experiments (5× higher in Reference [69]), which results in overall increased magnetic loading of the sample. Additionally, in Liao et al., the alginate-coated MNPs were incubated only for 30 min with the cells while, in the research from Taratula et al., the poly(maleic anhydride-alt-1-octadecene)- poly(ethylenimine)-poly(ethileneglycol)-coated MNPs were incubated with the cells for 12 h, which suggests an increased uptake capability for alginate-coated MNPs.

Many of the in vitro studies on MHT for cancer treatment do not refer to the concept of intracellular hyperthermia introduced by Gordon et al. [2], in the sense that AMF is applied right after adding the magnetic materials to the cells or without previously removing the extracellular or unbound MNPs. In those studies, the outcome, though sometimes impressive [70] and still dependent on the reached temperature and (time of) exposure to AMF, may be due to the heating of the surrounding environment and, therefore, not necessarily dependent on the direct interaction of MNPs and cells [70,71,72,73,74,75]. On the other hand, Blanco-Andujar and co-workers have recently reported magnetically-induced cell death due to intracellular heating, while excluding the heating effects of the local environment [11].

#### 2.2.4. Administration Route

For in vivo (and clinical) MHT studies, the route of administration of the NMs, along with their coating, contributes to differential distribution and accumulation of the NMs, and a balance needs to be found between the effective tumor treatment and the appearance of side effects due to off-target accumulation in vital organs such as the liver, spleen, and lungs. Many different administration routes have been explored in in vivo MHT studies (see Table A3), but there seems to be a preference for intra-tumoral (IT) and intravenous (IV) administration.

The IT administration of MNPs seems to be generally considered a more invasive procedure than the IV counterpart and it is known to result in uneven distribution of the MNPs in the tumor, which can lead to a significant temperature difference across the tumor [76]. This can be minimized by multiple site IT injection [77] or by magnetic targeting of MNPs after IV injection, i.e., using an external magnet to concentrate the MNPs in the area of interest [76,78]. Magnetic targeting, combined with antibody targeting and the EPR effect, have been reported to allow the tumor to be specifically heated [60]. Among the advantages of IT administration of MNPs are the less complex formulation usually required and that, in general, it results more effectively than the IV route [78]. Additionally, it opens the possibility to treat tumors where MNPs do not accumulate to sufficient amounts after IV injection.

In the case of IV administration of MNPs, a general dilution effect of the blood and the tumor volume should be considered as well as the limited injection volume. Additionally, the majority of the MNPs tends to accumulate in organs such as the spleen or the liver [79], and only a small fraction of the injected material will accumulate in the tumor, which may limit MHT efficiency [80]. Xie et al. confirmed that one single IV injection dose of MNPs does not result in accumulation of enough magnetic material to produce tumor heating [81]. Therefore, six repeated IV injections were performed, which was followed by 15 AMF cycles of 30-min each. This was still not enough to produce a reassuring result. Other authors have previously reported on the challenges of effective MNPs’ delivery to the entire tumor with active targeting [53,82]. The heat efficiency of the NM plays a crucial role in this scenario, which means that elevated in vivo MHT efficiency is still possible after IV administration [83]. A major advantage of IV administration of targeted MNPs for MHT is the possibility of equally treating tumor metastasis. Furthermore, while the accumulation of MNPs in the brain is very limited [79], the IV route for the injection of MNPs for treating brain cancer is discouraged. The natural proneness of MNPs to accumulate in the liver after IV injection [82,83] makes this organ an appealing target for MHT cancer therapy. It is interesting to notice, however, that, in most of the studies, the IT administration route is the one selected by the authors [45,77,84,85,86,87,88].

### 2.3. The Alternating Magnetic Field Component

#### 2.3.1. AMF Power

The AMF parameters to be used for the MHT treatment are usually established in advance before the first in vitro and in vivo studies. For that purpose, the heating performance of the MNPs is routinely assessed by subjecting them to different combinations of field amplitude and frequency for different periods of time [75,89]. The heating power of MNPs is commonly described by the specific absorption rate (SAR, expressed in W·g^−1^), which is also known as a specific loss power. This is the quantification of power dissipation of magnetic NMs under a specific AMF:

(1) $$SAR=\frac{C}{m_{Fe}}\frac{\Delta T}{\Delta t}$$

where C is the specific heat capacity of the sample, *m_Fe_* is the iron mass per unit volume, and Δ*T*/Δ*t* is the initial slope of the temperature (T) vs. time (t) curve [89]. Care must be taken, however, when comparing SAR values because they are affected by a number of parameters such as size, size distribution, chemical composition, and concentration of the NM, but also the amplitude (*H*) and frequency (*f*) of the applied AMF [90]. The product of these two variables, the *Hf* factor, correlates with the heating power and provides a quantitative index of the heating potential for a given AMF protocol. To avoid damaging the healthy tissues due to electromagnetic radiation (e.g., eddy currents), a threshold *Hf* of 5 × 10^9^ A∙m^−1^∙s^−1^ has been established [47,76]. Yet, this threshold is overcome in at least half of the MHT studies addressed in this paper (Table 1). Another factor to be considered is the use of different MHT setups, as the coil dimensions and the range of field amplitude and frequencies may vary, which, therefore, results in distinct SAR values [32]. Additionally, the MHT application protocol may vary between groups. Makridis et al. have addressed the impact of the AMF field conditions on MHT efficiency by testing two different NMs (Cobalt-Fe and Manganese-Fe) under two distinct MHT protocols (single pulse versus multiple pulse) to induce osteosarcoma cell death [91]. The Mn-Fe showed better MHT efficiency than the Co-Fe under the same AMF, which clearly exemplifies the impact of higher SAR values in the MHT efficiency. The heating rate and the heating location were considered the dominant factors to explain the enhanced efficiency of the multiple pulse protocol over the single pulse protocol.

#### 2.3.2. Reached Temperature and Time of Exposure

**Figure 4:**
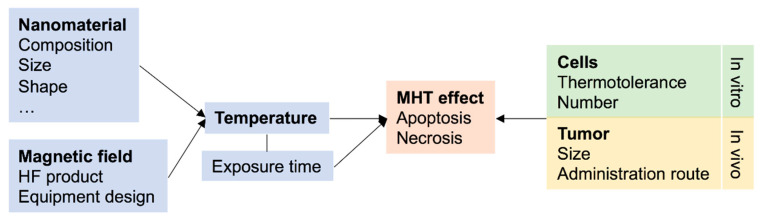
Main parameters contributing for the magnetic hyperthermia (MHT) effect in in vitro and in vivo scenarios.

The reached temperature is a crucial element to be disclosed in an MHT study due to the clear impact it may have in the observed outcome and cell death pathway. Still, nearly 30% of the studies herein addressed do not communicate such information. The temperature reached in an MHT study depends on the characteristics of the NMs and the magnetic field. In turn, the hyperthermia-derived effect will depend not only on the reached temperature but also on the type of cells and the time of exposure to heat (Figure 4) [92].

In in vitro studies, the sample’s temperature is usually measured using an optical fiber submerged in the cell culture medium. Infrared thermal imaging cameras are also used both in vivo and in vitro [80,83,93,94], which displaying a color-code image that correlates with the temperature reached in a specific area. Less conventional, and still under development, techniques include, for example, the use of fluorescence anisotropy-based thermoprobes, which are bioconjugates of dyes and proteins with increased thermo-sensitivity comparatively to the individual molecules [95]. 

At a tissue level, a rise in temperature to around 42 °C results in an increased tumor blood supply, which may be beneficial for the simultaneous delivery of chemotherapeutic agents. This enhances their anti-tumor effect [96]. Also, increased blood flow leads to increased oxygenation, which is a factor known to enhance radio-sensitivity [97]. On the other hand, an augment in temperature to above 42 °C decreases the tumor’s blood flow, while increasing blood flow in the normal tissues, which results in a lower dissipation rate and, therefore, a faster temperature rise in tumors, compared to normal tissues [98]. At a cellular level, biophysical and metabolic differences render cancer cells more susceptible to elevated temperatures than normal cells [20]. This fact implies the existence of a temperature range for which it is possible to kill cancer cells with minimal effects in normal cells, which is a major concern when using hyperthermia for cancer treatment.

Studies have demonstrated that the time of exposure to the AMF has an influence on the cell survival rate and clonogenic activity [9,10,75]. However, time only plays a role in case a damaging temperature is reached. In fact, a 1 °C drop within the temperature range of 42.5 to 47 °C can be compensated by duplicating the time of exposure, but, for temperatures below 42.5 °C, the time of exposure needs to be extended significantly more [9].

Court and co-workers found a positive correlation between cytotoxic effects, the reached temperature and the time of exposure, with 45 °C for 30 min inducing a drastic decrease in cell viability (below 10%) [10]. Under milder conditions, between 41 and 43 °C, the cytotoxic effects were intensified by combining the MHT with HSP70 inhibition, either by the HSPA6 gene knock-down, or by inhibiting HSP70 function using 2-phenylethyenesulfonamide. Slightly higher temperatures might be necessary in an in vivo scenario, as complete tumor regression mostly occurs when magnetic thermoablation is performed at temperatures higher than 45 °C [45,83,99]. This sometimes requires a higher NM dose and may result in adverse effects, such as bleeding and infection [99]. Still, some studies reaching temperatures above 50 °C did not report complete tumor regression, which may derive from the short-term exposure to such a high temperature, the bigger size of the tumor, or the different thermal susceptibility of the xenographs [17,84].

The number of MHT cycles also has an impact on the observed outcome, especially in in vivo studies (Table A3), where the number of cycles may vary from 1 to 15. The use of different cells, NM, and MHT protocols, makes it impossible to directly infer the influence of the number of cycles on the MHT efficiency. However, Zhang et al. reported that a lower number of MHT cycles resulted in apoptosis of cancer cells, and, consequently, tumor recurrence. On the other hand, a single cycle producing magnetic thermoablation led to cell necrosis and resulted in complete tumor regression [99]. This suggests that the number of MHT cycles becomes a trivial parameter in the cases where thermoablation is achieved.

#### 2.3.3. Apoptosis or Necrosis?

The ability to produce effective cell death in conditions of mild hyperthermia (41 to 43 °C) is much lower than in thermoablation conditions (at a temperature above 45 °C). Mild hyperthermia can induce apoptosis, but the effects of heat may be reversible due to the induction of HSP expression, which will counteract the heat-induced effects (thermotolerance) [47]. In the case of thermoablation, heat-induced protein denaturation, cytoskeleton, and membrane disruption, and altered deoxyribonucleic acid (DNA) conformation (among other molecular effects) most likely induce a necrotic type of cell death [9]. Table 2 describes the main characteristics of mild hyperthermia versus thermoablation.

### 2.4. Assessment of MHT Efficiency

#### 2.4.1. Time-Point after MHT

The efficiency of the implemented MHT protocol can be assessed immediately or sometime after the treatment. While the first approach may provide useful information, it does not guarantee the long-term preservation of the observed results. The heat insult may cause a sudden deregulation in cellular metabolism, which can be recovered if the death stimulus is removed and the conditions are favorable [9,11,25]. Sometimes, the actual cytotoxic effect may not be correctly evaluated until hours or even days after the treatment [11,61,100]. This is particularly relevant in studies where apoptotic temperatures are reached, instead of necrotic ones, as some of the surviving cells will be able to multiply and regrow the tumor. As an example, the viability of Jurkat cells exposed to a 1 h-MHT treatment that reached ~43 °C increased when the evaluation was performed 72 h, as compared to the 24 h after-treatment [61]. On the other hand, a full kill was observed when the treatment reached ~45 °C, which prevented cell recovery and would, expectedly, avoid tumor re-growth. Therefore, even though such treatments are often considered very efficient, there is still a margin for the cancer cells to grow back the tumor when full kill is not achieved.

#### 2.4.2. Most Commonly Used Tests

3-(4,5-dimethylthiazol-2-yl)-2,5-diphenyltetrazolium bromide (MTT) reduction and trypan blue exclusion are the most frequently used tests to assess cell viability in in vitro MHT studies (Table A2 and Table A3). The MTT assay measures the ability of NAD(P)H-dependent oxidoreductase enzymes in viable cells to metabolize the tetrazolium compound to a purple formazan [101]. This provides information of the momentary metabolizing activity, which can be transitorily impaired following the heat insult but can recover in case the conditions become favorable [9], and particularly if the cells exhibit a shorter doubling time [102]. Additionally, care must be taken in the use and interpretation of the MTT assay results since NMs can recognizably interfere with optical detection methods [103]. On the other hand, the trypan blue exclusion assay is based on the ability of viable cells to exclude the dye due to intact cell membranes [104]. Cells that are stained by trypan blue have, therefore, a damaged membrane, which is an irreversible type of cytotoxicity.

**Figure 5:**
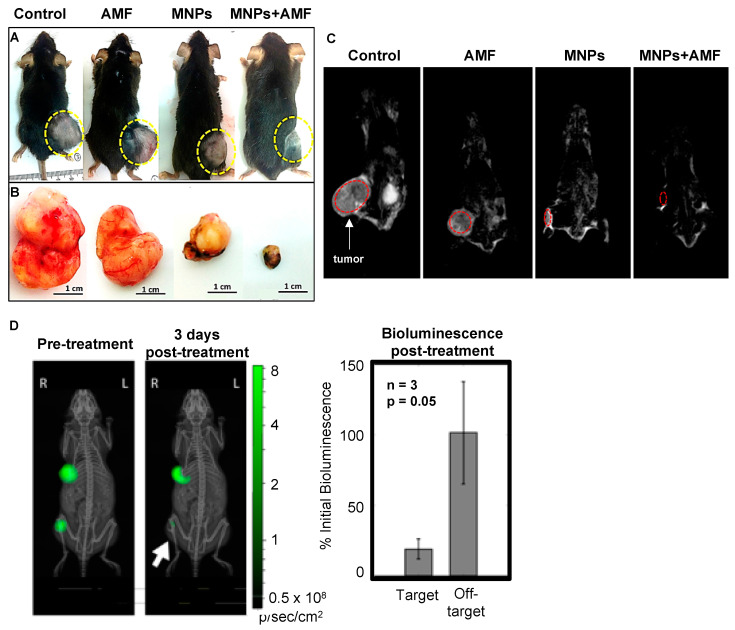
Strategies to evaluate heating effects in vivo. (**A**) Tumor volume in control and treated mice 30 days after treatment. (**B**) The tumors were excised from the animals for an easier-sized comparison. (**C**) Magnetic resonance imaging (MRI) also allows control of tumor size before and after treatment. Adapted with permission from Reference [23]. Copyright 2017 Elsevier. (**D**) Using luciferase-modified cell lines to generate the tumors, it is possible to track the bioluminescence signals and correlate them with tumor size. Adapted with permission from Reference [106]. Copyright 2018 American Chemical Society.

In case of in vivo studies, MHT efficiency is often described in terms of suppression of tumor growth or, more desirably, complete tumor elimination, by measuring the size or the weight of the remaining tumor after treatment (Table A3, Figure 5A,B). Taking advantage of the magnetic properties of MNPs, some authors use them as contrast agents, not only for diagnostic purposes but also to evaluate treatment efficiency with magnetic resonance imaging (MRI, Figure 5C) [23,56,105]. Alternatively, generating the tumor with luciferase-expressing cancer cells equally allows tracking tumor size using bioluminescence (Figure 5D) [106]. While suppressing tumor growth is an encouraging result, only complete tumor elimination can prevent tumor re-growth [19,45,83,87,89]. Therefore, in vivo MHT studies, approaching either mono-therapeutic or combinatorial schemes should aim at complete tumor elimination for more permanent results.

## 3. Remarks and Perspectives

The development of new magnetic NMs and the constant need to develop effective cancer therapies have considerably raised the interest of the scientific community in MHT studies during the past decade. Even though MHT has been approved by the European Medicines Agency for treating glioblastoma multiforme [97], the use of MHT to clinically treat cancer is still in its very infancy. This may be linked to the fact that different research groups used significantly different MHT protocols, which made it challenging or even impossible to draw comparisons between MHT experiments. Therefore, to help in the future implementation of MHT studies, we compiled the most relevant parameters to consider for the outcome of an MHT assay. These primarily concern the choice of (1) the biological component, (2) the NM, (3) the MHT setup and conditions, and (4) the assessment of the MHT outcome.

The susceptibility of cancer cells to heat is variable and, therefore, a certain MHT protocol may be quite effective in killing certain cells and not others [8]. Similarly, the efficiency of the procedure may differ between in vitro and in vivo settings [10]. Using cellular models that most closely resemble an in vivo scenario should be, therefore, highly supported. The thermal susceptibility of the cell line, the number of cells used in the study, and the 2D or 3D configuration of the cells are details that will impact the results.

The size, the shape, and the coating of the NM are crucial characteristics directly influencing the outcome of an MHT experiment [32,107]. In an in vivo setting, these specifications, together with the presence of a targeting moiety, also affect the NMs distribution, which provides an additional indirect contribution to the MHT outcome [31,44]. Even though NMs that self-regulate the hyperthermia temperature have been suggested as a way to minimize damaging healthy tissues, these were barely tested in vitro [48]. Therefore, more studies are necessary to support their potentiality. In general, biocompatible NMs with increased SAR values are preferred because they are more efficient heat mediators. However, the dependence of SAR on the *Hf* product brings about the need to control the AMF parameters to stay below the biologically-safe threshold. A fine-tuning is, therefore, necessary to balance the AMF power with the NMs concentration to obtain efficiency while avoiding undesired toxicity.

The outcome of the MHT experiment strongly depends on how and when it is evaluated [61,100]. Time is needed for some types of toxicity to manifest (e.g., apoptosis), but also to investigate whether recurrence (or tumor re-growth) will happen in standard conditions. In this sense, a standardization of the time-point at which the outcome of the MHT experiment would be checked would be an important consideration. This, alone, would immediately indicate whether a certain protocol yields more promising results than another. Importantly, most of the in vivo MHT studies focus on the effectiveness of the treatment by tracking the outcome months after MHT. However, little is mentioned about the fate of the NMs in the body, which is an issue that should be more considered in future in vivo studies.

The difficulty to find effective MHT protocols that could be applied in clinical conditions resulted in the suggestion to use MHT as an adjuvant to other therapies [10,93,94,108]. Most studies show that this results in a more efficient treatment, yet does not exclude the occurrence of side effects that usually accompany the individual therapy.

To summarize, in our opinion, the starting point for the implementation of an MHT (pre)clinical experiment should be the maximum tolerable AMF power threshold. Next, the selection of a biocompatible NM with fine-tuned characteristics, including SAR, would be essential. The protocol should aim at localized heating, using targeting antibodies or magnetic gradients that attract the NMs to a specific location, or focus the AMF in a certain area, which keeps healthy tissues unheated. The NMs developed for this end seem to be, in general, well tolerated but the dependence of the heat production on the NM concentration/payload and the AMF parameters still needs to be established and clarified. For preclinical tests, the biological component should resemble the clinical situation as much as possible. Advantage should be taken on the natural differences between normal and cancer cells, namely their thermotolerance and their magnetic loading ability. Since the future will increasingly rely on the use of human-based, more advanced 3D in vitro models, the assessment of the MHT efficiency should be standardized as the first step to allow the comparison between the outcomes of different MHT protocols, which possibly leads to more competence in the transition from the preclinical to clinical application of MHT.

## Figures and Tables

**Table 1:** Summary of the *Hf* product values of the studies addressed in this review.

Number of Studies	*Hf* > 5 × 10^9^ A∙m^−1^∙s^−1^	*Hf* < 5 × 10^9^ A∙m^−1^∙s^−1^	No Reported T
72	37	24	17
%	51.4%	33.3%	23.6%

NOTE: For 15.3% of the studies herein considered, it was not possible to calculate the *Hf* product. T—temperature.

**Table 2:** Main characteristics and outcomes of mild hyperthermia and thermoablation conditions.

	Mild Hyperthermia		Thermoablation
T (°C)	41–43		>45
Tumor tissue	Increased blood supply Increased oxygenation		Decreased blood supply Lower dissipation rate
Tumor cells	Protein denaturation ER stress response activation Inhibition of DNA repair APOPTOSIS	Induction of HSP THERMOTOLERANCE	Protein denaturation Cytoskeleton rearrangement Membrane disruption Altered DNA conformation NECROSIS

ER—endoplasmic reticulum, DNA—deoxyribonucleic acid, and HSP—heat shock protein.

## Appendix A

**Table A1:** Conditions used, and outcome observed, in in vitro studies using magnetic hyperthermia (MHT) alone or combined with other therapeutic regimens organized by the cell line in alphabetical order.

Cell Line	MNPs			Inc. Time (h)	[Fe] Sample	MNPs Excess Removal	Magnetic Field			Reported Temp (°C)	% Cell Viability (h after MHT) Method	Obs.	Ref.
	Coating	Size nm (Technique)	Initial Conc. (g·L^−1^)				F (kHz)	H (Ka m^−1^)	Time (min)				
4T1 + C166 + MEF	D-mannitol	~150 (SEM)	0.1 (LD), 1 (HD)	24	NR	Yes	300	55	60	NR	Significant increase cell death/damage (72) Sytox^®^ Blue	Co-culture of three distinct cell types to simulate a 3D in vitro model of triple negative breast cancer lung metastasis.	[14]
A2780cp20; HeyA8	Carboxy methyl dextran	~60 and ~200 (bimodal, DLS)	0.5 (Fe)	0	NA	No	245	24 or 36	30 or 60	41, 43 or 45	41 °C = cell viability unaffected; 43 °C: <40; 45 °C: <10; (48) TB	Combined with HSP70 inhibition (HSPA6 siRNA or PES) potentiated MHT at 41 °C (HSPA6: 15–25% cell viability HeyA8, 25–50% A2780cp20; PES: <40% cell viability both cell lines).	[10]
A549; MDA-MB-231	Myristic acid + pluronic F127	12 ± 3 (TEM); ~185 (DLS)	1.5 (Fe_3_O_4_)	0	NA	No	386	6	5, 15, 30	43–46	~60, ~30, ~10, resp. (2) LDH	Similar efficiency for both cell lines; no colonies formed two weeks after MHT; MHT for cancer stem cell elimination.	[75]
BV2	PAA	36 ± 8 (TEM)	0.1	24	NA	Yes	560	23.9	30	46	25 (4.5), TB	Cell pellets used for treatments. Comparable efficiency between MHT and water bath hyperthermia. Suggest the use of microglial cells as MNP carriers to treat glioma with MHT.	[12]
C6	Aminosilane	NR	10 (Fe)	0	NA	No	305	23,9	30	41–43	80 (10 min); 0 (0.5) Live/dead^®^	Tumor-on-a-chip to mimic glioblastoma	[15]
CT-26	PEG	The SPIONs are ~20 (TEM)	0.1	2	NA	No	293	12.57	15	~40	90, 85, 74, (48, 72, 96), MTT	3 cycles of MHT days 1, 2 and 3. MTT on days 2, 3 and 4. Incorporation of DOX resulted in synergistic (45%, 30%, and 18% cell viability at 48, 72 and 96 h, resp.). Theranostic formulation (great MRI contrast).	[109]
DU-145	Starch	~108 (DLS)	0.015, 0.075, 0.1, 0.15 (Fe)	16–24	5, 70, 105, 199 pg/cell, resp.	Yes	150	88	30	37–49.1	<10 for T > 43 °C (10–14 days) clonogenic survival assay	PDL as facilitator of MNPs uptake; different reached temperature according to cell pellet size and iron/cell.	[13]
DX3	Citric acid	~17 (TEM); ~66 (DLS)	0.5	12	Susp: 210-400; Adhe: 315 pgFe_3_O_4_/cell	Yes	Susp: 911 or 950; Adhe: 950	Susp: 6.6; 10.5; 12; 14.7 or 16.1; Adhe: 10.8	120	40 to 50	Susp:~55 to ~5; Adhe: 3 (24) Annexin-V/Pi	No normal cells control; experiments in adhe cells suggest the occurrence of cell specific events, supporting individual cell hyperthermia.	[11]
ESO26, OE21, NIH-3T3	NA	NA	NA	24	NR	NA	270	29.4 or 34.7	10 sec, day 1 and 2	45	CPI of ~1.3 for both cancer cell lines (12) WST-8	Poly(NIPAAm-CO-HMAAm)/polyurethane coating a nitinol stent; NIH-3T3 only for biocompatibility studies; CPI drops to 0.1 when combined with 5-FU and PTX.	[110]
HeLa	Phospho lipid-PEG	~14 (TEM)	0.1 (Fe)	0	NA	No	355	24	60	43–46	~55 (24) MTT	When using DOX-MNPs = 32% cell viability.	[111]
HepG2	NA	689 ± 155 (DLS)	0.2 mg	0	NA	No	750	0.8	30	43	~50 (24) WST1	MHT increased intracellular ROS levels and DNA damage.	[112]
HT-1080	Dextran	~225 (DLS)	NR (drug conc 147 nM)	NR	NA	No	250	27.9	30	~43.5	69 (48) TB	When PTX-loaded magnetic liposomes are used for MHT cell viability decreases to 28%.	[113]
Jurkat	PMAO-PEG	12, 13, 16 (TEM) ~20 (DLS)	0.6, 0.6 or 0.49, resp.	0.25	NA	No	373	14	15	~38.5, ~40 and ~43.5,	90, 75, and 40, resp. (0.5) ATP levels	Optimization of MNP size and polydispersity to enhance MHT response at a selected AMF frequency.	[33]
KB	Pullulan acetate	~10 (TEM); 25.8 ± 6.1 (DLS)	NR	0	NA	No	100	10.4	20	45 or 47	45 °C = 45, 47 °C = 20 (24) MTT	L929 (normal) cell viability without AMF application = 90%.	[22]
KB	PEG	19 ± 3 (TEM) 37 ± 11 (DLS)	1 (Fe)	24	25–170 pg/cell 1.3–5.4 g/L	Yes	110	20	60	43	0–75 (NR) TB	2–4gFe/L to reach 40–45 °C = 50% cell viability; at 5gFe/L reached 65 °C = 0% cell viability.	[68]
MCF-7	Triethylene glycol: triethanol amine	~44 (TEM)	0.5 (Fe)	0	NA	No	240	89	60	45	25 (48) MTT	Nanoclusters also suitable for MRI in vivo for the un-clustered 10 nm MNPs = 40% cell viability.	[72]
MCF-7	Chitosan	20–30 (TEM)	1	0	NR	NR	267	24	120	44–45	60 (1) TB	L929 (normal) = 93% cell viability.	[25]
MCF-7	Terephthalic acid	10 ± 2 (TEM) 150.9 ± 0.5 (DLS)	1	0.25	NA	No	751.5	10.9	60	45	0 (24) TB	Highly effective MHT but without MNP removal, so not intracellular hyperthermia.	[70]
MCF-7	Oleic acid + aspartic acid	11 (TEM)	1.5 or 2.5	3	NA	No	265	26.7	20	NR	17 for the 1.5 and 23 for the 2.5 (24)% of sub-G1 cells	The aspartate coated MNPs exhibited enhanced interaction with cells and superior killing effects when compared to pristine MNPs.	[114]
MCF-7	Polyamidoamine dendrimer	10 ± 4 (TEM); 120 (DLS)	0.5	2	120 pg/cell	No	300	12	120	NR	36.7 (0) MTT	Normal HDF1 cell viability = 63.5% when treated similarly.	[28]
MCF-7; MCF-7/ADR	mPEG−PCL	~100 (TEM and DLS)	0.2 (MNCs), 0.1 (MNPs)	0	NA	No	114	115	15	NR	10 (24) MTT	MnFe_2_O_4_/MNC vs. Mn_0.6_Zn_0.4_Fe_2_O_4_/MNC, both with similar MHT efficiencies, in both cell lines. Use low AMF exposure times.	[74]
MDA-MB-231	PEG bis(amine)	~15 (TEM)	0.2	5	NA	No	500	37.4	60	43 ± 1	75 (NR) WST-8	When using GdTx-MNPs for MHT = 36% cell viability (GdTx as sensitizer to MHT).	[115]
MDA-MB-231	Chitosan	~18 (TEM), ~90 (DLS)	1.5 (Mn and Fe)	0	NA	No	307	50 (then to 20 or 35)	30	42 or 52, resp.	22.5 and 18, resp. (24) Annexin-V/Pi	24, 48, and 72h incubation w/ MNPs yielded 100, 112 and 146 pg Fe/cell, MHT42 = apoptosis, MHT52 = necrosis.	[73]
MDA-MB-468, Caco-2, A2780	Carboxy methyl dextran	69 ± 4 (TEM)	3.8	0	NA	No	233	29.4 or 34.7	30	43 or 45, resp.	MDA-MB-468 43 °C = 50, 45 °C = 30, Caco-2 43 °C = 35, 45 °C = 15, A2780 43 °C = 25, 45 °C = 5 (48) TB	Reported enhanced effects of bortezomib in combination with MHT (cell viability <20% in all cases).	[8]
MG-63	Sodium oleate	25–40 (TEM)	5	0	NA	No	186	17	60	37–49	>90 up to 43 °C and 54 at 47 °C (0) MTT	MHT on average 16% more efficient than water-based hyperthermia. The short time after MHT for assessing cytotoxicity does not allow to quantify apoptotic effects.	[116]
MIA-PaCa-2	PLGA	1:1:10–204 1:1:20–245 (DLS)	1	NR	NA	No	0.323	90	180	NR	1:1:10–25, 1:1:20–50 (0) TB	MHT to potentiate chemotherapy with HSP90 inhibitor 17AAG. Elevated cytotoxicity L929 cells (normal).	[24]
SaOS-2	Citric acid	NR	1	48	0.5 g/L	Yes	765	20–24	10 (2 cycles with 48 h distance)	MnFe: 45 CoFe:41–45	Single pulse: MnFe: 70, CoFe: 70 Multiple pulse: MnFe: 35, CoFe 70, (0) TB	Testing 2 binary ferrites in single vs. multiple pulse for MHT. Multiple pulse resulted in enhanced cytotoxicity specifically in cancer cells. Low cytotoxicity was observed for the normal cell line (3T3-L1) treated similarly.	[91]
SH-SY5Y	PEI	NR	0.1	24	NR	Yes	570	3.98–23.9 (to control target temp)	30	37–51	37 °C: 90; 40 °C: 75, 42 °C: ~50, 44 °C: ~40; 46 °C: ~25; 48 °C: ~10, 50 °C: <5 (6) TP	MHT induced higher cytotoxicity than water bath for the same target temp. Cytotoxic effects increased with increased time-point after treatment.	[100]
SKOV-3	Liposomes	150 (TEM), 200 (DLS)	0.5–5 mM (Fe)	1–4	~20 pg/cell (for the 5 mM Fe)	Yes	700	24	30	NR	10 (12) AlamarBlue	When combined with PDT = 0% cell viability, missing MNP concentration of reported cell viability data.	[93]
SKOV-3	Gallol-PEG	~20 (TEM)	0.2 mM (Fe)	2	~6 pg/cell	Yes	520	20	10	38–40	75 (NR) AlamarBlue	When combined with PTT = 15% cell viability and T = 50 °C.	[94]
SMMC-7721	NA	17 ± 2 (TEM)	1	0	NA	No	50	34	40	42, 44 and 44.3	75.4, 61.5 and 53.6 (24) MTT	Application of a static magnetic field to limit the heating to a restricted area.	[71]
U87	PEG	177±17 (DLS) 153 (TEM)	0.3 (Fe)	4	NA	No	Varied to keep temp	Variable to keep temp	30	Multiple MHT:44 °C; single MTA: 50 °C	NR (0) Annexin-V/Pi	Increased number of apoptotic cells with increasing number of MHT cycles. Gradual progression from apoptosis to necrosis from single to multiple MHT. Extensive necrosis for MTA.	[99]
U87	Methoxy-PEG-silane 500 Da	22.8 ± 3.3 (DLS)	0.7	NR	500 pg/cell (for 0.5 g·L^−1^)	Yes	99	12.33	25	63.5	Complete necrosis (NR) CCK-8	Resovist as a control reached 37.5 °C and induced no change in cell viability.	[45]
U87MG	PEG	50–100 (TEM)	NR	0	NA	No	750	16	120/ day x 4 days	43	44.5 (72) Annexin-V/Pi	No significant improvement was found when the nano-vectors were loaded with temozolomide	[46]
U87-EGFRvIII	PEI	77 ± 11 (DLS)	0.01	5	NR	Yes	225	5	45	44.1	60 (24) MTS; 80 in spheroids	Magnetofection to facilitate MNPs uptake; if combined with let-7a microRNA = 34% cell viability (45% in spheroids).	[117]

Conc.—concentration. Inc.—incubation. NA—not applicable. NR—not reported. resp.—respectively. LD—low dose. HD—high dose. susp—suspended. adhe—adherent. MTA—magnetic thermal ablation. TEM—transmission electron microscopy. DLS—dynamic light scattering. XRD—x-ray diffraction. MNCs—magnetic nanoclusters. MNPs—magnetic nanoparticles. HAP—hydroxyapatite. PES—2-phenylethynesulfonamide. PEG—poly(ethylene glycol). PEI—poly(ethylenimine). PLGA—Polylactic-co-glycolic acid. PMAO—poly(maleic anhydride-alt-1-octadecene). mPEG-PCL—monomethoxy-terminated poly(ethylene glycol)-b-poly-(ε-caprolactone). PDT—photodynamic therapy. PTT—photothermal therapy. CPI—cell proliferation index (cell number day 3 divided by cell number day 1). Cell viability tests—TB—trypan blue exclusion. MTT—3-(4,5-dimethylthiazol-2-yl)-2,5-diphenyltetrazolium bromide reduction. MTS—3-(4,5-dimethylthiazol-2-yl)-5-(3-carboxymethoxyphenyl)-2-(4-sulfophenyl)-2H-tetrazolium reduction. SRB—sulforhodamine B binding. WST—8-2-(2-methoxy-4-nitrophenyl)-3-(4-nitrophenyl)-5-(2,4-disulfophenyl)-2H-tetrazolium reduction.

**Table A2:** Conditions used, and outcome observed, in in vitro studies using targeted MHT alone or combined with chemotherapeutic agents.

Functionalization	Cell Line	MNPs			Inc. Time (h)	[Fe] Sample	MNPs Excess Removal	Magnetic Field			Reported T ( °C)	% Cell Viability (h after MHT)	Observation	Ref.
		Coating	Size (nm)	Initial Conc. (g·L^−1^)				F (kHz)	H (kA m^−1^)	Time (min)				
Anti- αβγ_3_^a^	MDA-MB231	PEG	30–35 (TEM)	520 μM (Fe)	6	NR	NR	480	10	15	44	7.3 (0) MTS	Cell viability results from targeting + DOX + MHT. Only MHT = 48%, MHT + DOX = 31%.	[56]
Anti-CD90 ^b^	Huh7	PEG	10–20 (TEM), 130 ± 4.6	0.34 (Fe)	1	NA	Yes	200	NR	60	44	30 (24) MTT	Thermosensitive magneto-liposomes. CD90+ separated from CD90− by MACS and then treated with MHT.	[87]
Anti-CXCR4 ^b^	Jurkat	Targ: dextran-PA; Non-targ: PAA	Targ: 250, Non-targ 18 (TEM)	Targ: 0.362; Non-targ: 0.396 (Fe)	Targ: 1; Non-targ 2	Targ: 122; Non-targ: 4.3; comb: 155 pg/cell	Yes	1st: 869 2nd: 554	1st: 20 2nd: 24	30 + 30	~43 or ~45	16 (when 43 °C); 0 (when 45 °C) (72) PB	Biphasic AMF to push temp to max and then stabilize it. MHT using targeted MNPs-only cell viability = 75%. Induction of necrosis is more effective than apoptosis.	[61]
Anti-CXCR4 ^b^	LN229 HK-2	Targ: dextran-PA; Non-targ: PAA	Targ: 250, Non-targ 18 (TEM)	Targ: 0.264; Non-targ: 0.260 (Fe)	Targ: 1; Non-targ 2.5	LN229: 108; HK-2: 38 pg/cell	Yes	1st: 869 2nd: 554	1st: 20 2nd: 24	30 + 30	LN229: 46.9; HK-2: 41.2	LN229: <10 and 0 HK-2: 75 and 80 (24 and 72) PB	Optimization of the MHT approach to tumor cells expressing lower levels of target receptor; HK-2 (normal) cells practically undamaged.	[21]
CREKA ^b^	A549	Dextran	5–13 (TEM);	3 (Fe_3_O_4_)	NA	NR	Yes?	292	58	30	NR	40 (48 & 72) Calcein-AM	Incubation suspended cells + NP. Reported additive effects of cysplatin to 20% cell viability after 72 h.	[118]
Dipeptide (Arg-∆Phe) ^b^	A549, NCI-H460, HLF-1 (normal)	NA	~146 (TEM); ~123 (DLS)	0.08	3?	A549 2.5 Non-targ, 1.9 Targ; NCI-H460 2.8 Non-targ, 3.23 Targ (µg/mL)	No	50	175 mA	180	NR	A549: Non-targ 59; Targ 72; NCI-H460: Non-targ 65; Targ 85; (24) Pi	Use pulsed electromagnetic field. No significant differences in cell viability between targeted and non-targeted MNPs. HLF-1 (normal) cells not affected by similar treatment.	[26]
Folate ^a^	MCF-7, G1	Carboxy methyl cellulose	100–150 (TEM); 80–200 (DLS)	2 and 4	NA	NR	NR	305	18	60	NR	20 (24) TB	If combined with 5-FU = 5% cell viability.	[119]
Folate ^b^	HeLa (FR + )	PEG	84.9 (TEM)	0.5	1	0.3 g/L	No	750	0.8	10	43–45	NR	Reported LDH values of 0.76 compared to 0.45 for untreated control. Normal human fibroblasts not affected.	[27]
Folic acid ^a^	HeLa	Poly acrylic acid	8–10 (TEM)	2	24	~250 pg/cell (24h inc. w/0.3 g L^−1^)	Yes	265	27	10	NR	65 (24) SRB	When using DOX loaded FA-MNPs = 50% cell viability, DOX-FA-MNPs + AMF = 10% cell viability.	[108]
Folic acid ^a^	SKOV3	PEG	120–140 (TEM)	0.5	NR	NR	NR	200	NR	30	NR	~14 (72) MTT	Magnetic thermosensitive liposomes are loaded with HSP90 inhibitor, 17AAG, combining chemotherapy with targeted-MHT	[57]
Galactose ^b^	HepG2	Alginate	109.1–146.9 (HD)	0.5	4	364.4 pg/cell	Yes	780	19	20	NR	5 (18) MTT	Only applicable for hepatic tumors.	[29]
Herceptin ^a^	SKBR3	APTES-PEG	NR	100 μg (Fe)	NR	NR	NR	100	~22	5	42	33 (48) TB	If combined with RIT cell viability = 3.3%. First study on combined use of RIT + DOX + targeted SPIONs to kill HER overexpressing cells.	[60]
Herceptin ^b^	SK-BR-3	Dextran	138 ± 7.6 (DLS)	28.6 pg Fe_3_O_4_/ cell	4	16.5 pg Fe_3_O_4_/ cell	Yes	360	9.6	30	42.5	25 (24) TB	Cell viability recovered after 5 days in culture. When AMF was repeated 24 h after, cell viability <10% after 5 days.	[120]
Herceptin ^a^	SK-BR-3	PLA-TPGS/ TPGS-COOH	155.2 ± 0.17 (DLS)	0.86; (177 ug Fe)	24	NR	NR	240	42	20 or 30	NR	30 (12) MTT	If combined with docetaxel = 10% cell viability.	[58]
Herceptin ^a^	MIAPaCa-2	PLGA	524 ± 9 (DLS)	0.1	48	NR	Yes	440	16.2	15	NR	NR (0)	Gemcitabine released with MHT, AO-EB staining showed late cell apoptosis/ necrosis and decreased Bcl2 and cyclin-D1 expression.	[23]
Hyaluronic acid ^a^	4T1	Polypyrrole	83.6 (DLS)	0.493 (Fe_3_O_4_)	24	NR	NR	635	30 A-	15	NR	CTR = 25 MHT = 14 (12) ALDH ^+^ cells	When Notch inhibitor is incorporated in the formulation ALDH^+^ cells = 9%, mammosphere cells: [CTR = 35 MHT = 17.5 MHT + Notch inhibitor = 9] ×10^4^ cells. Claim effective elimination of cancer stem cells.	[59]
Hyaluronic acid ^b^	SCC7, NIH3T3 (normal)	None vs. PEG	100–272 (DLS)	0.1	1	NR	NR	368	1	10	42	~30 (24) MTS	CD44- cell viability unchanged under similar MHT protocol. No differences in MHT outcome between PEG-coated or non-coated MNP.	[121]
iRGD ^a^	U87-EGFRvIII; MDA-MB-231	PEI + PEG	46.8 ± 2.3 (DLS)	0.02	24	NR	Yes	300	5	45	NR	40 (48) MTS	Magnetofection to facilitate MNPs uptake. MHT as an enhancer of peptide therapeutics.	[122]
LHRH peptide ^a^	A2780/AD	PMAO + PEI + PEG	~40 (TEM) ~90 (DLS)	0.015 (Fe)	12	14.9 pg/cell	Yes	393	33.5	30	44	5 (48) Calcein-AM	Similar cell viability achieved with DOX loaded LHRH-MNPs combined with MHT at 40 °C.	[69]

Conc.—concentration. Inc.—incubation. NA—not applicable. NR—not reported. targ—targeted. non-targ—non-targeted. comb—combined. TEM—transmission electron microscopy. DLS—dynamic light scattering. MACS—magnetic-activated cell sorting. RIT—radio-immunotherapy. CSC—cancer stem cells. LHRH—luteinizing hormone–releasing hormone. APTES—3-aminopropyltriethoxy silane. PA—protein A (from *Staphylococcus aureus*). PEG—poly(ethylene glycol). PEI—poly(ethylenimine). PMAO—poly(maleic anhydride-alt-1-octadecene). PLA-TPGS—poly(lactide)-D-a-tocopheryl poly(ethylene glycol) succinate. TPGS-COOH—carboxyl group-terminated TPGS. RIT—radio-immunotherapy. Cell viability tests—ALDH—Aldehyde dehydrogenase. MTS—3-(4,5-dimethylthiazol-2-yl)-5-(3-carboxymethoxyphenyl)-2-(4-sulfophenyl)-2H-tetrazolium reduction. MTT—3-(4,5-dimethylthiazol-2-yl)-2,5-diphenyltetrazolium bromide reduction. SRB—sulforhodamine B binding. TB—trypan blue exclusion. ^a^—Combination with other therapies. ^b^—Mono-therapeutic context.

**Table A3:** In vivo studies using passive or active targeted anti-cancer MHT.

Cell Line (Number of Cells)	In Vivo Model	Initial Tumor Size	MNPs			MNP. Inj. Mode	Magnetic Field					T (°C)	Outcome	Observation	Ref.
			Coating	Size (nm)	Initial Amount		Time after Inj.	F (kHz)	H (kA m^−1^)	Time (min)	Cycles				
Human breast cancer MCF-7 (2 × 10^6^)	BALB/c female mice	50 mm^3^	-	97.85 ± 0.74 (DLS)	NR	IV	0	423	10	30	5	42	Delayed tumor progression	AMF to favor tumor accumulation. Triple effect: MHT, cell-penetrating peptides to increase DOX uptake. Thermo-responsive DOX release.	[16]
Human breast cancer MCF-7 (2 × 10^6^)	BALB/c female mice	100 mm^3^	-	NR	NR	IV	0	423	10	30	5	42–43	Delayed tumor progression, increased tumor accumulation, augmented c-Myc silencing	AMF to favor tumor accumulation. Triple effect: MHT, cell-penetrating peptides to increase siRNA delivery, thermo-responsive siRNA-CPP release.	[123]
Human breast cancer MDA-MB-231 (NR)	NS (nude mice)	100 mm^3^	PEG bis (amine)	~15 (TEM)	75 µg	IT	0	500	37.4	30	1	43	Incomplete tumor regression day 8, tumor regrowth day 12	When using GdTx-MNPs for MHT = tumor eliminated within eight days.	[115]
Human breast cancer SKBR3 (3 × 10^5^)	BALB/c female mice	0.2 cm^3^	APTES-PEG	NR	0.5 mgFe	IV	48	100	~22	15	2	NR	Tumor volume inhibitory rate day 28 = 85%, nearly complete tissue necrosis	Permanent magnet for magnetic delivery w/o detectable damage to surrounding tissues. Trastuzumab-conjugated, radiolabeled, DOX-loaded MNPs suitable detection by MRI or SPECT (targeted MHT + radio + chemo + imaging). WBC decreased by 23% day 28.	[60]
Human chronic myeloid leukemia K562/A02 (1 × 10^7^)	BALB/c mice	950 ± 150 mm^3^	Oleic acid + Pluronic F-127	18.4 ± 1.8 (TEM)	22 µg MCL/g body weight	IT	0	219	10.5–310	40	1	~42	40% decrease relative tumor volume	If combined with DNR + 5-BrTet = 80% decrease relative tumor volume and decreased P-gp expression.	[124]
Human epidermoid carcinoma A431 (5 × 10^6^)	SCID female mice	200–400 mm^3^ (calc.)	PLGA-*b*-PEG	77.8 ± 2.1 (DLS)	400–800 µL of 4.5 g Fe_3_O_4_/L, days 16, 17, 18, 19, and 22	IT	0	173	25	30	5	5–6 inc.	1.7 × inc. MS	hEGFR-targeted MNPs. Observed increased temperature in subsequent treatments. Higher accumulation in liver and lungs than tumor after IV injection of MNPs.	[80]
Human fibrosarcoma HT-1080 (1 × 10^6^)	Swiss female mice	8–15 mm diameter	Dextran	225 ± 45 (DLS)	SD = 1, DD = 2 (mg MNPs)	IT	0	250	27.9	30	SD = 3, DD = 5	42.5	SD: Significantly slower tumor growth both w/ and w/o PTX, DD: w/ PTX significantly different from w/o PTX	CTR mice sacrificed on day 12 due to tumor burden; SD sacrificed day 16; DD sacrificed day 22 (days after 1^st^ dose); treatment considered harmless to the body.	[113]
Human glioblastoma U251 (1 × 10^7^)	BALB/c female mice	NR	-	NR	50 mM	SC	0	280	335.4 A_rms_	60	1	43.1	Tumor size reduction: Fe(Salen) + MHT = 80–90%, but not significantly different from Fe(Salen) only = 50%	The tumor model was injected in the mice leg due to limited injection volume in the mice brain; sacrifice day 28.	[125]
Human glioblastoma U87MG (1 × 10^7^)	BALB/c nude mice	100–150 mm^3^	PEG	177 ± 16.9 (DLS)	MHT: 3.5 µgFe/µL, MTA: 8.7 µgFe/µL	IT multi	0	389	19.5	25	MHT: 1, 2, 3, or 4. MTA: 1	MHT: 45 (43-44^th^ cycle). MTA: 53.1	MHT: Significant tumor regression after 3 or 4 cycles, but tumor recurrence if only 3 cycles are performed. MTA: complete tumor regression	Sacrifice day 25. MTA lead to serious bleeding and infection.	[99]
Human glioblastoma U87MG (1 × 10^7^)	BALB/c nude mice	100–150 mm^3^	PEG	SPIONs: 18; hydrogel: 519 ± 141 (TEM)	2.9 µgFe/µL	IT multi	0	366	13.3	60	2	43 ± 1	Significant inhibition of tumor growth specially after 2 cycles. 100% survival rate	Sacrifice day 25; TRAIL-loaded hydrogel; 2 MHT cycles enhanced TRAIL-induced cytotoxicity; neither kidney nor liver damage; long-term MRI imaging.	[105]
Human hepatocellular carcinoma Hep3B (NR)	NS (nude mice)	1000 mm^3^	Methoxy-PEG-silane 500 Da	22.8 ± 3.3 (DLS)	100 µL of 1.15 g/L	IT	0	99	12.33	15	1	50.2	Complete tumor elimination 2 days after MHT (bioluminescence imaging)	Mice under observation for 1 month: no tumor regrowth, no severe side effects.	[45]
Human hepatocellular carcinoma HepG2 (1 × 10^6^)	BALB/c female mice	0.5 cm diameter	PEI	20–30 (TEM)	1 mg/cm^3^ tumor	IT	0	230	NR	60	2	42–44	50% or 90% reduction tumor mass 28 days after MHT, resp. MHT and MHT + gene therapy	Combination of MHT with gene therapy targeting α-fetoprotein in hepatocarcinoma.	[88]
Human hepatocellular carcinoma HepG2 (2 × 10^6^)	BALB/c female nude mice	0.3–0.5 cm^3^	PEI	15–20 (TEM)	5 mg of 10 g/L	IT multi	24 h	230	NR (30 A)	60	3	42–45	Decrease tumor mass 6 weeks after MHT = 77%, MHT + radio + gene = 94%	MNPs functionalized w/ anti-α-fetoprotein antibody. Multimodality treatment combining MHT, radio and gene therapy. No side effects on liver, kidney and no inhibition of hematopoiesis.	[77]
Human hepatocellular carcinoma HepG2 (1 × 10^7^)	BALB/c male nude mice	~0.4 cm^3^	PRO	15–20 (TEM)	500 mg/mL	IT multi	24 and 48 h	110	8.8	30	2	43	MHT-only and gene therapy-only did not block tumor growth. The combination of both caused the tumor to shrink.	Sacrifice day 30 after injection; gene therapy = delivery of the TNFα gene.	[86]
Human hepatocellular carcinoma Huh7 CD90^+^ (2 × 10^4^)	NOD/ SCID mice	600 mm^3^	PEG	10–20 (TEM). 130 ± 4.6 (DLS)	NR	IT	24 h	200	NR	60	3	NR	27.3 ± 9.8% complete tumor regression 70 days after injection	When using Anti-CD90-MNPs = 78 ± 19.1% complete tumor regression 70 days after injection. Rectal T < 40 °C.	[87]
Human hepatocellular carcinoma Huh7 (2 × 10^4^)	Athymic nude female mice	NR	Silica	NR	932 μg/mL	IT	0	750–800	NR	30	1	~43	Attenuation of tumor growth compared to control, but still tumors grew from day 0.	Heat-induced release of ansamitocin (chemo + MHT), sacrifice day ~10,A follow-up treatment would be necessary to sustain inhibitory effect on tumor growth.	[85]
Human hepatocellular carcinoma SMMC-7721 (1 × 10^6^)	NS (nude mice)	~450 mm^3^	PLGA	NA	NR (50 μL of 20% Fe)	IT	0	626	NR (28.6 A)	~2	1	52	Tumor ablation and elimination 5 days after MHT observed in synergy with DOX release upon heating.	MHT alone produces tumor ablation with residual growth 8 days after treatment.	[84]
Human lung squamous carcinoma A549-Luc (1 × 10^6^)	Fox Chase SCID female mice	0.5 × 10^6^ photons/s	Myristic acid + pluronic F127	369 ± 34 (DLS)	NR	IH	7 days	386	6	30	1	NR	Reduction in tumor growth rate over 1 month after MHT. Reduced tumor weight.	No significant side effects. Inhalation led to higher tumor MNPs accumulation than other organs.	[126]
Human ovarian cancer HeyA8 or A2780cp20 (1 × 10^6^)	Athymic nude mice	35–113 mm^3^	Carboxy methyl dextran	~60 and ~200 (bimodal, DLS)	5 mgFe/cm^3^	IT or IP	4 h	245	23	30	3	43	Reduction in tumor growth (volume and weight) is enhanced by the number of MHT treatments.	Combination with HSP70 silencing tumor growth is inhibited.	[10]
Human ovarian carcinoma OVCAR-3 (1 × 10^6^)	Balb/c nu/nu female mice	100 mm^3^	Carboxy dextran	77 (DLS)	300 µgFe/mL (5 × 10^5^ SPION-labeled MSC)	SC	NA	1050	8 (10 mT)	20	4	41.5; 40.8; 39.7; 38.2, resp.	No difference in tumor growth (tumor volume similar to control).	SPION-labeled MSC injected simultaneously with tumor cells. Magnetic heating effect decreased with cycles: heat-induced MSC death and clearance?	[18]
Human ovarian carcinoma SKOV3 (5 × 10^6^)	BALB/c female nude mice	NR	PEG	~130 (TEM)	10 mgFe/Kg	IV	NR	200	NR	30	1 (or 4?)	NR	MHT = 63%. MHT + 17-AAG = 68%; MHT + 17-AAG + FA = 85% tumor weight inhibition rate.	4 IV injections of MLS. Not clear whether AMF is applied only one time or one time after each injection (total 4 times).	[57]
Human pancreatic cancer MIAPaCa-2 (1.5 × 10^7^)	B6.CB17-Prkd^scid^/szJ mice	>50 mm^3^	PLGA	524 ± 9 (DLS)	2 mg/Kg GCT equivalent	IT	0	440	16.2	15	10	6 inc.	86% reduction tumor volume after 30 days. Reduced expression of Bcl-2 and cyclin-D1	Herceptin-targeted nanospheres containing fluorescent IONP and GCT, tested MHT and MRI,	[23]
Mouse breast cancer 4T1 (5 × 10^6^)	BALB/c female mice	50–80 mm^3^(calc.)	PEG	54.6 (DLS)	6 × 30 µg Fe/g body weight (every other day)	IV	30 min	390	2.6	30	15	Passive: 38.7–42.5; Active: 39.6–44.1	59% apoptotic cells and delayed tumor growth	Passive vs. active targeting (ανβ3 integrin—targeted MNCs); studied bioaccumulation in the main organs, combined MRI and MHT, no clear toxicity	[81]
Mouse breast cancer 4T1 (5 × 10^6^)	BALB/c nude mice	~80 mm^3^	Polypyrrole	83.6 (DLS)	18.64 mgFe/Kg	IT	0	635	30 A	15	4	45 (calc.)	Slower tumor growth, significantly lower tumor weight, decreased number of CD44 ^+^ cells: targeted-MHT = 57%, targeted-MHT + chemo = 33%	Theranostic tool: chemotherapy mediated by Notch inhibitor + targeted-MHT + dual-mode MRI and photoacoustic imaging	[59]
Mouse breast cancer 4T1 (5 × 10^6^)	BALB/c female mice	~80 mm^3^ (calc.)	PEG	30–45 (TEM)	0.25 mgFe/ 100 mm^3^	IT	60 min	480	10	15	1	NR	Tumor volume inhibition day 16 —MHT = 18%, MHT + DOX = 88%, targ-MHT + DOX = 89% w/ absence of metastasis	Targeting of ανβ3 integrin contributes for the absence of metastasis. Enhanced MRI-T2 contrast.	[56]
Mouse colon carcinoma CT-26 (1 × 10^5^) or mouse melanoma B16F10 (1.25 × 10^5^)	BALB/c or C57BL/6 mice	5 × 6 mm (2 tumors/ mouse)	BNF-Starch	100 (TEM)	140 µg Fe	ID	0	167.5	36–44	20 or 30	1	42.5–43 for 30 min; 44.5–45 for 20 min	43 °C = Complete elimination of treated CT26 tumor in BALB/c in 5 days + untreated tumor grew slower, incomplete elimination of treated B16 tumor in C57BL/6 + untreated tumor grew slower, 45 °C = complete elimination of treated B16 tumor in C57BL/6 + no effect in the untreated tumor	Hyperthermia-induced immunologic response at 43 but not at 45 °C. Immunologic response was not observed for Lewis lung carcinoma tumors, even at 43 °C. rectum T = 35.5–37 °C	[19]
Mouse Lewis lung cancer (2 × 10^6^)	C57/BL6 male mice	0.8 ± 0.1 cm diameter	NR	NR	15 mg magnetic fluid	IT	24 h	150	Variable to keep temp	30	1	~43	Tumor volume decreased ~38% in MHT treated group and ~71% in MHT + IL-2 group	Improved treatment for Lewis lung cancer in mice when MHT is combined w/ regular IL-2 injections.	[64]
Mouse squamous cell carcinoma SCC7 (2 × 10^5^)	NCr nude mice	~150 mm^3^	PEG	11.3 ± 2.3 (TEM) 23.8 ± 0.1 (DLS)	1.7 g Fe/Kg body weight	IV (tail vein)	24 h	980	38	2	1	60	Complete tumor ablation in 78–90% cases	Muscle w/ MNPs T = 42 °C; Muscle *w*/*o* MNPs T = 36 °C; Liver uptake > tumors. 16:1 MNP ration tumor to non-tumor.	[83]
Rabbit carcinoma VX-2 (1 × 10^5^)	New Zealand white rabbits	1.4 cm^3^	PLGA	NR	100 µL of 30% Fe_3_O_4_	IT	0	626	NR (28.6 A)	3	1	72.3 ± 2.2	MHT-only = Incomplete tumor ablation, if combined w/ cisplatin release = residual tumor elimination by day 21	Larger tumor and animal model. possible to track by ultrasound or computer tomography.	[17]

Inj.—injection. NR—not reported. NS—not specified. DD—double dose. SD—single dose. MTA—magnetic thermoablation. inc.—increase. -Luc—Luciferase transfected. TEM—transmission electron microscopy. DLS—dynamic light scattering. MACS—magnetic-activated cell sorting. MNPs—magnetic nanoparticles. MCLs—magnetite cationic liposomes. DNR, daunorubicin. GCT—Gemcitabine. 5-BrTet—5-bromotetrandrine. LHRH—luteinizing hormone–releasing hormone. MS—mean survival. multi—multisite. APTES—3-aminopropyltriethoxy silane. PEG—poly(ethylene glycol). PEI—poly(ethylenimine). PLGA—poly(D,L-lactide-co-glycolide). PRO—protamine sulfate. Administration routes: IT—intra-tumor. IV—intravenous. IH—inhalation. ID—intradermal. SC—subcutaneous.
